# Development of Analytical Model to Describe Reflectance Spectra in Leaves with Palisade and Spongy Mesophyll

**DOI:** 10.3390/plants13223258

**Published:** 2024-11-20

**Authors:** Ekaterina Sukhova, Yuriy Zolin, Kseniya Grebneva, Ekaterina Berezina, Oleg Bondarev, Anastasiia Kior, Alyona Popova, Daria Ratnitsyna, Lyubov Yudina, Vladimir Sukhov

**Affiliations:** 1 Department of Biophysics, N.I. Lobachevsky State University of Nizhny Novgorod, 603950 Nizhny Novgorod, Russia; n.catherine@inbox.ru (E.S.); uchebnayap.zolin@gmail.com (Y.Z.); grebneva.kseniya01@mail.ru (K.G.); nastay2903@bk.ru (A.K.); silverkumiho@mail.ru (A.P.); dasha-lola1997@mail.ru (D.R.); lyubovsurova@mail.ru (L.Y.); 2 Department of Biochemistry and Biotechnology, N.I. Lobachevsky State University of Nizhny Novgorod, 603950 Nizhny Novgorod, Russia; berezina.kat@gmail.com; 3 Department of Botany and Zoology, N.I. Lobachevsky State University of Nizhny Novgorod, 603950 Nizhny Novgorod, Russia; olegbond353@rambler.ru

**Keywords:** analytical reflectance model, Beer–Bouguer–Lambert law, dicot higher plants, Kubelka–Munk theory, leaf reflectance spectra, palisade mesophyll, spongy mesophyll

## Abstract

Remote sensing plays an important role in plant cultivation and ecological monitoring. This sensing is often based on measuring spectra of leaf reflectance, which are dependent on morphological, biochemical, and physiological characteristics of plants. However, interpretation of the reflectance spectra requires the development of new tools to analyze relations between plant characteristics and leaf reflectance. The current study was devoted to the development, parameterization, and verification of the analytical model to describe reflectance spectra of the dicot plant leaf with palisade and spongy mesophyll layers (on the example of pea leaves). Four variables (intensities of forward and backward collimated light and intensities of forward and backward scattered light) were considered. Light reflectance and transmittance on borders of lamina (Snell’s and Fresnel’s laws), light transmittance in the palisade mesophyll (Beer–Bouguer–Lambert law), and light transmittance and scattering in the spongy mesophyll (Kubelka–Munk theory) were described. The developed model was parameterized based on experimental results (reflectance spectra, contents of chlorophylls and carotenoid, and thicknesses of palisade and spongy mesophyll in pea leaves) and the literature data (final R^2^ was 0.989 for experimental and model-based reflectance spectra). Further model-based and experimental investigations showed that decreasing palisade and spongy mesophyll thicknesses in pea leaves (from 35.5 to 25.2 µm and from 58.6 to 47.8 µm, respectively) increased reflectance of green light and decreased reflectance of near-infrared light. Similarity between model-based and experimental results verified the developed model. Thus, the model can be used to analyze leaf reflectance spectra and, thereby, to increase efficiency of the plant remote and proximal sensing.

## 1. Introduction

Terrestrial plants can be affected by numerous environmental factors influencing their growth and development, productivity, physiological processes, and content of biochemical compounds. Early-revealing changes in the characteristics of plants play an important role in their agricultural cultivation and ecological monitoring and require the development of methods of remote sensing [1,2]. Optical methods, including RGB imaging, fluorescence imaging, thermal imaging, and imaging of spectral characteristics of reflected light, are effective tools for plant remote sensing [3,4] because they are non-invasive, relatively simple, and fast.

Particularly, multispectral imaging, which is based on measuring reflectance in several narrow spectral bands, and hyperspectral imaging, which is based on measuring reflectance spectra, are widely used for plant remote sensing [3,4]. It is known that leaf reflectance in narrow spectral bands can be related to specific plant characteristics, including the content of photosynthetic pigments [5,6,7], water content [8,9,10], nitrogen content [11,12], leaf area index and biomass [12,13,14,15], photosynthetic processes and their stress changes [16,17,18,19,20], etc. Calculation of reflectance indices, which are mainly dimensionless indicators based on reflectance at two or three wavelengths, is used to improve these relationships and to increase efficiency of estimation of plant characteristics [3,4].

However, relationships of specific reflectance indices in leaves to specific plant characteristics can be varied [3]; for example, this variability was shown [21,22,23] for photochemical reflectance index based on reflectance at 531 and 570 nm. It is known that this index is dependent on the acidification of the chloroplast lumen (through transitions in the xanthophyll cycle [17,19] and through the chloroplast shrinkage [20]) and, therefore, should be potentially related to photosynthetic processes. Moreover, our earlier meta-analysis of experimental studies [23] showed that average linear correlation coefficients between values of photochemical reflectance indices and photosynthetic parameters (the photosynthetic light use efficiency, potential quantum yield of photosystem II, and non-photochemical quenching of chlorophyll fluorescence) are about 0.5–0.6.

There are several possible reasons for the variability of relationships of reflectance at specific wavelengths (and, therefore, reflectance indices) to plant characteristics [3]. First, a photosynthetic pigment composition, which can be dependent on plant species [24,25], individual development [11,26,27,28], and environmental conditions [24], strongly influences reflectance spectra (through changes in spectra of the light absorption in the leaf lamina). Second, differences in the leaf anatomy can also increase variability of reflectance spectra [3]. It is known that reflectance of visible light and near-infrared light (NIR) is different on adaxial and abaxial leaf surfaces [29,30,31], which is caused by different optical properties of palisade and spongy mesophylls in dicot plants [29,32]. Reflectance is also dependent on the leaf thickness [25,32,33] and presence of hairs [34]. Third, leaf orientation (angle between the leaf surface and incident light) [25,29] and their fluttering under the wind [35] can also modify reflectance spectra. Fourth, changes in phenological stages of plants and their senescence strongly influence reflectance spectra [6,26,36,37]. The influence can have complex reasons, including modification of content of photosynthetic pigments and leaf orientation [37], that is, it can be caused by mechanisms, which are noted above.

Thus, variability of leaf reflectance spectra, which decreases relationships of parameters of these spectra to plant characteristics, can limit analysis of results of the plant remote sensing and requires the development of tools to minimize these limitations. Mathematical models of optical characteristics of leaves, which consider content of photosynthetic pigments and anatomical structure, can be potentially used as these tools. In accordance with [38], there are several types of models to describe the optical properties of leaves, namely, Beer–Bouguer–Lambert law-based models, plate models, N-flux models (or Kubelka–Munk theory-based models), compact spherical particle models, radiative transfer theory-based models, stochastic models, and ray tracing models.

Kubelka–Munk theory-based models, which are widely used to analyze light reflectance and transmittance in leaves and canopy [39,40,41,42,43,44,45,46], are relatively simple (analytical solutions can be derived) and often provide accurate descriptions of the optical properties of plants. The models consider two (forward and backward) [39,41,43,44,45,46] or four (forward collimated, forward scattered, backward collimated, and backward scattered) [40,42] light flows. Particularly, the Kubelka–Munk theory-based model can be used to derivate light absorption coefficients from reflectance spectra of canopy [44] and leaf [45,46,47] or to analyze light-dependent chloroplast movements [48]. It should also be noted that description of plant leaf can be difficult due to several layers with different optical properties (mainly, palisade and spongy mesophyll layers, which are typical for dicot plants) [38]. There is the Kubelka–Munk theory-based model (with forward and backward light flows) [41] describing different layers in plant leaves; this study shows that Kubelka–Munk theory-based models can be potentially used to describe reflectance of leaf with different layers.

Moreover, simple Beer–Bouguer–Lambert law-based models can be used at weak light scattering [38]. Considering the low light scattering coefficient in the leaf palisade mesophyll [49], these models are probable to be effective to describe the optical properties of the layer. Using Beer–Bouguer–Lambert law-based models for the palisade mesophyll should simplify an analytical description of light reflectance in leaves.

Thus, the current study was devoted to the development, parameterization, and verification of an analytical model to describe leaf reflectance spectra in dicot higher plants (with palisade and spongy mesophyll layers), which was based on both the Kubelka–Munk theory and the Beer–Bouguer–Lambert law as well as on other widely used approaches (particularly the Snell’s and Fresnel’s laws). The model can be potentially used as the tool for analysis of results of the plant remote sensing on the basis of reflectance measurements and for the development of new indicators to estimate plant characteristics.

## 2. Description of the Mathematical Model of Light Reflectance in Leaves

### 2.1. Basic Structure and Variables of the Model

**Figure 1:**
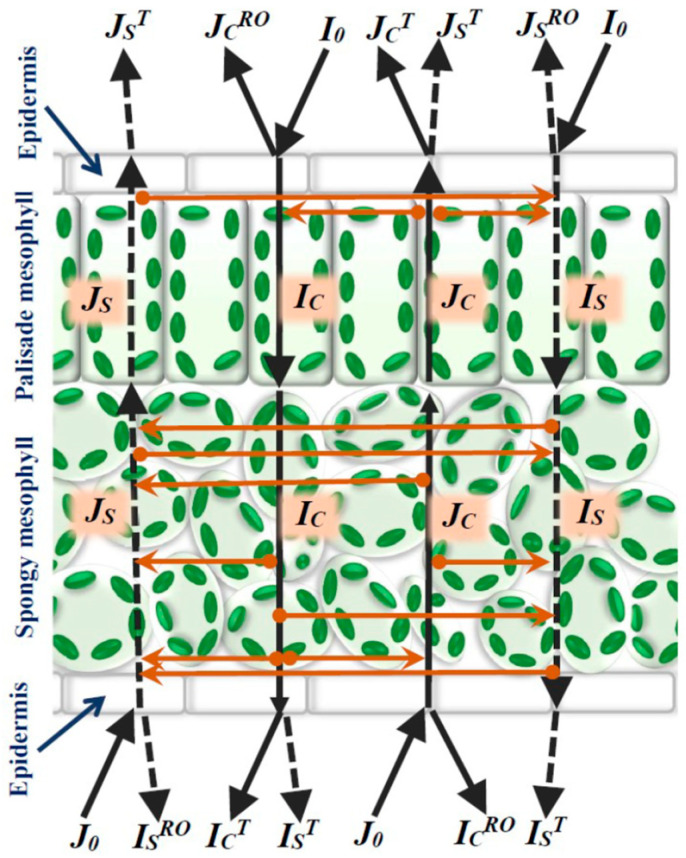
The scheme of light flows in the model of reflectance and transmission in the leaf of a dicot plant. Black continuous lines show forward light flows. Black dotted lines show backward light flows. Orange lines show transformation between light flows. *I_C_* is the forward collimated light, *I_S_* is the forward scattered light, *J_C_* is the backward collimated light, and *J_S_* is the backward scattered light. *I_0_* and *J_0_* are intensities of the forward and backward collimated light directed to adaxial and abaxial leaf surfaces, respectively (the incident light). *J_C_^RO^* and *I_C_^RO^* are intensities of the collimated light reflecting from adaxial and abaxial leaf surfaces in air. *J_S_^RO^* and *I_S_^RO^* are intensities of the scattered light reflecting from adaxial and abaxial leaf surfaces in air. *J_C_^T^* and *I_C_^T^* are intensities of the collimated light transferring from leaf to air across adaxial and abaxial surfaces. *J_S_^T^* and *I_S_^T^* are intensities of the scattered light transferring from leaf to air across adaxial and abaxial surfaces.

**Figure 2:**
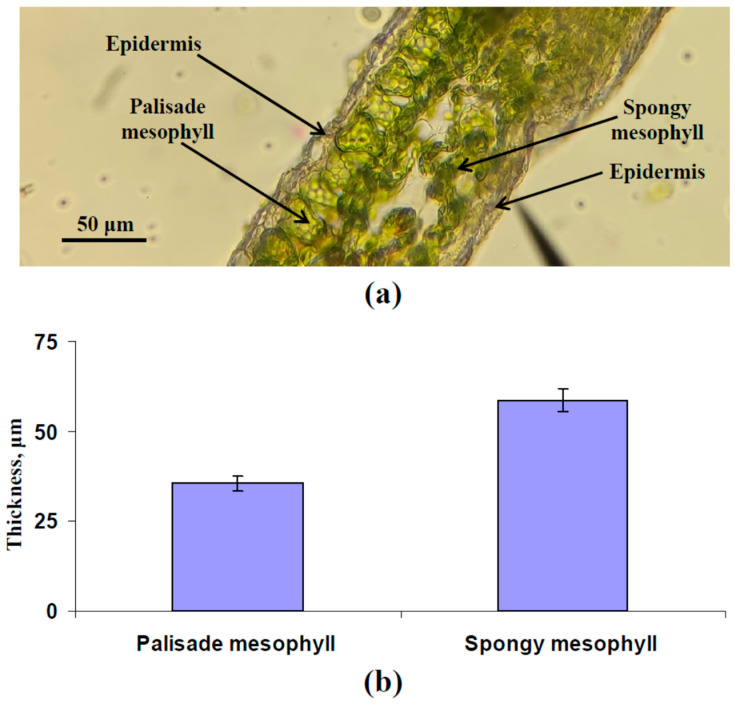
Image of pea leaf cross-section (**a**) and average thickness of palisade (*h*) and spongy (*l*) mesophyll (*n* = 6) (**b**). The second mature leaf was used. Pea plants were cultivated for 16 days under the moderate light intensity.

In accordance with Figure 1, the leaf was assumed to be an optical system, including two main optical layers, namely, the palisade mesophyll layer with high light absorption ($a_{P}$) and low light scattering ($s_{P}$) coefficients and the spongy mesophyll layer with high light absorption ($a_{Sp}$) and high light scattering ($s_{Sp}$) coefficients [49]. Epidermal layers had small thickness and weak color (Figure 2a) and were not considered in the model. Borders “air-leaf” and “leaf-air” were additionally described in the model. Four variables were considered in the current study (Figure 1), including intensities of the forward collimated light (*I_C_*), forward scattered light (*I_S_*), backward collimated light (*J_C_*), and backward scattered light (*J_S_*) [40,42].

### 2.2. Equations Describing Light Reflectance and Transmittance on Borders “Air-Leaf” and “Leaf-Air”

It was assumed that there were two types of transmittances of the collimated light across each border (“air-leaf” and “leaf-air”): (i) across a smooth surface and (ii) across a rough surface. The fraction of the rough surface (*F_S_*) was the model parameter; the fraction of the smooth surface was calculated as 1-*F_S_*.

Relationships between the angles of incidence and refraction were described by Equations (1) and (2), which were based on Snell’s law:

(1) $$\beta_{I1}=\arcsin\left(\frac{n_{O}}{n_{I}}\sin\left(\beta_{O1}\right)\right)$$

(2) $$\beta_{I2}=\arcsin\left(\frac{n_{O}}{n_{I}}\sin\left(\beta_{O2}\right)\right)$$

where *n_O_* and *n_I_* are the refractive indices in air (*n_O_* = 1) and in leaf (*n_I_* = 1.415 [50]); *β_O_*_1_ and *β_O_*_2_ are the angles of incidence on adaxial and abaxial leaf surfaces, respectively; and *β_I_*_1_ and *β_I_*_2_ are the angles of refraction under adaxial and abaxial leaf surfaces, respectively.

Fresnel’s law-based Equations (3) and (4) were used for the calculation of transmittance coefficients for the collimated light transfer from air to leaf across the smooth surfaces [51,52]:

(3) $${T_{Ic}}^{OI}=1-\frac{1}{2}\left[{\left(\frac{\sin\left(\beta_{O1}-\beta_{I1}\right)}{\sin\left(\beta_{O1}+\beta_{I1}\right)}\right)}^{2}+{\left(\frac{\tan\left(\beta_{O1}-\beta_{I1}\right)}{\tan\left(\beta_{O1}+\beta_{I1}\right)}\right)}^{2}\right]$$

(4) $${T_{Jc}}^{OI}=1-\frac{1}{2}\left[{\left(\frac{\sin\left(\beta_{O2}-\beta_{I2}\right)}{\sin\left(\beta_{O2}+\beta_{I2}\right)}\right)}^{2}+{\left(\frac{\tan\left(\beta_{O2}-\beta_{I2}\right)}{\tan\left(\beta_{O2}+\beta_{I2}\right)}\right)}^{2}\right]$$

where *T_Ic_^OI^* and *T_Jc_^OI^* are transmittance coefficients for the collimated light transfer from air to leaf on adaxial and abaxial surfaces, respectively. Thus, Equations (5) and (6) were used for the calculation of the intensity of the collimated light transmittance into the leaf across adaxial and abaxial surfaces (*I_C_*(0) and *J_C_*(*h* + *l*), respectively):

(5) $$I_{C}(0)=I_{0}\cdot \left(1-F_{S}\right)\cdot {T_{Ic}}^{OI}$$

(6) $$J_{C}(h+l)=J_{0}\cdot \left(1-F_{S}\right)\cdot {T_{Jc}}^{OI}$$

where *I_0_* and *J_0_* are the intensities of the forward and backward collimated light directed to adaxial and abaxial leaf surfaces (the incident light), respectively; *h* is the thickness of the palisade mesophyll layer; and *l* is the thickness of the spongy mesophyll layer.

Equations (7) and (8) were used for the calculation of the intensity of the collimated light reflecting from adaxial and abaxial leaf surfaces in air (*J_C_^RO^* and *I_C_^RO^*, respectively):

(7) $${J_{C}}^{RO}=I_{0}\cdot \left(1-F_{S}\right)\cdot \left(1-{T_{Ic}}^{OI}\right)$$

(8) $${I_{C}}^{RO}=J_{0}\cdot \left(1-F_{S}\right)\cdot \left(1-{T_{Jc}}^{OI}\right)$$

Equations (9) and (10) were used for the calculation of the transmittance coefficients for collimated light transfer from leaf to air:

(9) $${T_{Ic}}^{IO}=1-\frac{1}{2}\left[{\left(\frac{\sin\left(\beta_{I1}-\beta_{O1}\right)}{\sin\left(\beta_{I1}+\beta_{O1}\right)}\right)}^{2}+{\left(\frac{\tan\left(\beta_{I1}-\beta_{O1}\right)}{\tan\left(\beta_{I1}+\beta_{O1}\right)}\right)}^{2}\right]$$

(10) $${T_{Jc}}^{IO}=1-\frac{1}{2}\left[{\left(\frac{\sin\left(\beta_{I2}-\beta_{O2}\right)}{\sin\left(\beta_{I2}+\beta_{O2}\right)}\right)}^{2}+{\left(\frac{\tan\left(\beta_{I2}-\beta_{O2}\right)}{\tan\left(\beta_{I2}+\beta_{O2}\right)}\right)}^{2}\right]$$

where *T_Ic_^IO^* and *T_Jc_^IO^* are the transmittance coefficients for the collimated light transfer from leaf to air on abaxial and adaxial leaf surfaces, respectively. Equations (9) and (10) could not be solved at *β_I_*_1_ and *β_I_*_2_, which were more than about 45° because light was fully reflected in this case; the case was considered to describe transmittance and reflectance of the scattered light. It should be additionally noted that *T_Jc_^IO^ = T_Jc_^OI^* and *T_Ic_^IO^ = T_Ic_^OI^* for the collimated light.

Equations (11) and (12) were used for the calculation of the intensity of the collimated light transferring from leaf to air across adaxial and abaxial surfaces (*J_C_^T^* and *I_C_^T^*, respectively):

(11) $${J_{C}}^{T}=J_{C}\left(0\right)\cdot \left(1-F_{S}\right)\cdot {T_{Jc}}^{IO}$$

(12) $${I_{C}}^{T}=I_{C}\left(h+l\right)\cdot \left(1-F_{S}\right)\cdot {T_{Ic}}^{IO}$$

Equations (13) and (14) were used for the calculation of the intensity of the collimated light reflecting from adaxial and abaxial leaf surfaces in the lamina (*I_C_^RI^* and *J_C_^RI^*, respectively):

(13) $${I_{C}}^{RI}=J_{C}\left(0\right)\cdot \left(1-F_{S}\right)\cdot \left(1-{T_{Jc}}^{IO}\right)$$

(14) $${J_{C}}^{RI}=I_{C}\left(h+l\right)\cdot \left(1-F_{S}\right)\cdot \left(1-{T_{Ic}}^{IO}\right)$$

Transmittance coefficients for the collimated light transfer across the rough surfaces and for the scattered light transfer across both smooth and rough surfaces were described by Equations (15)–(18):

(15) $${T_{Is}}^{OI}=\frac{2}{\pi }\int_{0}^{\pi /2}{T_{Ic}}^{OI}d\beta_{O1}$$

(16) $${T_{Js}}^{OI}=\frac{2}{\pi }\int_{0}^{\pi /2}{T_{Jc}}^{OI}d\beta_{O2}$$

(17) $${T_{Is}}^{IO}=\frac{2}{\pi }\int_{0}^{\pi /2}{T_{Ic}}^{IO}d\beta_{I1}$$

(18) $${T_{Js}}^{IO}=\frac{2}{\pi }\int_{0}^{\pi /2}{T_{Jc}}^{IO}d\beta_{I2}$$

where *T_Is_^OI^* and *T_Js_^OI^* are the transmittance coefficients for the light transfer from air to leaf on adaxial and abaxial surfaces, respectively, and *T_Is_^IO^* and *T_Js_^IO^* are transmittance coefficients for the light transfer from leaf to air on abaxial and adaxial surfaces, respectively. It should be noted that *T_Is_^OI^* = *T_Js_^OI^* and *T_Is_^IO^* = *T_Js_^IO^*; thus, *T_s_^O^*^I^, equaling to *T_Is_^OI^* (=*T_Js_^OI^*) and *T_s_^IO^* equaling to *T_Is_^IO^* (=*T_Js_^IO^*) were used in the analysis. On basis of numerical calculation, it was shown that *T_s_^OI^* ≈ 0.866 and *T_s_^IO^* ≈ 0.469. These values were used for modeling.

Equations (19) and (20) were used for the calculation of the intensity of the scattered light transferring from air to leaf across adaxial and abaxial surfaces (*I_S_*(0) and *J_S_*(*h* + *l*), respectively):

(19) $$I_{S}(0)=I_{0}\cdot F_{S}\cdot {T_{s}}^{OI}$$

(20) $$J_{S}(h+l)=J_{0}\cdot F_{S}\cdot {T_{s}}^{OI}$$

Equations (21) and (22) were used for the calculation of the intensity of the scattered light reflecting from adaxial and abaxial leaf surfaces in air (*J_S_^RO^* and *I_S_^RO^*, respectively):

(21) $${J_{S}}^{RO}=I_{0}\cdot F_{S}\cdot \left(1-{T_{s}}^{OI}\right)$$

(22) $${I_{S}}^{RO}=J_{0}\cdot F_{S}\cdot \left(1-{T_{s}}^{OI}\right)$$

Equations (23) and (24) were used for the calculation of the intensity of the scattered light transferring from leaf to air across adaxial and abaxial surfaces (*J_S_^T^* and *I_S_^T^*, respectively):

(23) $${J_{S}}^{T}=J_{C}\left(0\right)\cdot F_{S}\cdot {T_{s}}^{IO}+J_{S}\left(0\right)\cdot {T_{s}}^{IO}$$

(24) $${I_{S}}^{T}=I_{C}\left(h+l\right)\cdot F_{S}\cdot {T_{s}}^{IO}+I_{S}\left(h+l\right)\cdot {T_{s}}^{IO}$$

Equations (25) and (26) were used for the calculation of the intensity of the scattered light reflecting from adaxial and abaxial leaf surfaces in the lamina (*I_S_^RI^* and *J_S_^RI^*, respectively):

(25) $${I_{S}}^{RI}=J_{C}\left(0\right)\cdot F_{S}\cdot \left(1-{T_{s}}^{IO}\right)+J_{S}\left(0\right)\cdot \left(1-{T_{s}}^{IO}\right)$$

(26) $${J_{S}}^{RI}=I_{C}\left(h+l\right)\cdot F_{S}\cdot \left(1-{T_{s}}^{IO}\right)+I_{S}\left(h+l\right)\cdot \left(1-{T_{s}}^{IO}\right)$$

Equations (27) and (28) were used for the calculation of the total intensities of light flows from adaxial and abaxial leaf surfaces to air (*J_out_*^1^ and *I_out_*^1^, respectively):

(27) $${J_{out}}^{1}={J_{C}}^{RO}+{J_{C}}^{T}+{J_{S}}^{RO}+{J_{S}}^{T}$$

(28) $${I_{out}}^{1}={I_{C}}^{RO}+{I_{C}}^{T}+{I_{S}}^{RO}+{I_{S}}^{T}$$

where “1” shows that these light intensities were calculated on the basis of the first iteration of the light propagation through leaf (see Section 2.5 for details).

### 2.3. Equations Describing Light Transmittance in the Palisade Mesophyll Layer

It is known that the palisade mesophyll layer has the low light scattering coefficient ($s_{P}$ = 5 cm^−1^) in comparison to the spongy mesophyll layer ($s_{Sp}$ = 1000 cm^−1^) [49]. It is known that the thickness of the palisade mesophyll layer (*h*) is often less than 70–80 μm [49,53,54,55]. It means that the probability of the primary light scattering in the palisade mesophyll layer is less than 3–4%; that is, a description of this scattering is not necessary to describe the light propagation through the layer.

As a result, we used Beer–Bouguer–Lambert law [38] as the basis of the description of the light propagation through the palisade mesophyll layer. Equations (29)–(32) were used for this description:

(29) $$I_{C}(x)=I_{C}(0)\cdot e^{-\frac{a_{P}}{\cos\left(\beta_{I1}\right)}x}$$

(30) $$J_{C}(x)=J_{C}\left(h\right)\cdot e^{-\frac{a_{P}}{\cos\left(\beta_{I2}\right)}\left(h-x\right)}$$

(31) $$I_{S}(x)=I_{S}(0)\cdot e^{-2a_{P}x}$$

(32) $$J_{S}(x)=J_{S}\left(h\right)\cdot e^{-2a_{P}\left(h-x\right)}$$

where *x* is the coordinate, $a_{P}$ is the light absorption coefficient, and “2” is the coefficient showing increase in the light absorption (and scattering) for the scattered light (on the basis of pathlength averaging over a hemisphere [42]).

Based on these equations, Equations (33)–(36), which described the light intensity on borders of the palisade mesophyll layer, were derived:

(33) $$I_{C}(h)=I_{C}(0)e^{-\frac{a_{P}}{\cos\left(\beta_{I1}\right)}h}$$

(34) $$J_{C}(0)=J_{C}\left(h\right)\cdot e^{-\frac{a_{P}}{\cos\left(\beta_{I2}\right)}h}$$

(35) $$I_{S}(h)=I_{S}(0)\cdot e^{-2a_{P}h}$$

(36) $$J_{S}(0)=J_{S}\left(h\right)\cdot e^{-2a_{P}h}+{J_{S}}^{Add}$$

where *J_C_*(0) and *I_C_*(*h*) are the intensities of collimated backward and forward light on upper and lower borders of the palisade mesophyll layer, respectively; *J_S_*(0) and *I_S_*(*h*) are intensities of scattered backward and forward light on upper and lower borders of the palisade mesophyll layer, respectively; and *J_S_^Add^* is the additional scattered light. *J_S_^Add^* is caused by the scattering (and changing light direction) of the scattered (*I_S_*(*x*)) and collimated (*I_C_*(*x*)) forward light in the palisade mesophyll layer. The *J_S_^Add^* should have low intensity; however, it can be important for the plant remote sensing based on measuring reflectance at red and light spectral bands, which have high light absorption.

We used Equation (37) to calculate *J_S_^Add^*:

(37) $${J_{S}}^{Add}=I_{C}(0)\cdot \frac{s_{P}\left(1-f\right)}{\cos\left(\beta_{I1}\right)}\int_{0}^{h}e^{-\frac{a_{P}}{\cos\left(\beta_{I1}\right)}x}\cdot e^{-2a_{P}x}dx+2I_{S}(0)\cdot s_{P}\left(1-f\right)\int_{0}^{h}e^{-2a_{P}x}\cdot e^{-2a_{P}x}dx$$

where $s_{P}$ is the light scattering coefficient in the palisade mesophyll layer and *f* is the asymmetry factor, which can describe the anisotropy of scattering (we assumed that *f* = 0.5; i.e., the asymmetry was absent). Equation (38) is the solution of Equation (37):

(38) $${J_{S}}^{Add}=I_{C}(0)\cdot \frac{s_{P}\left(1-f\right)}{a_{P}\left(1+2\cos\left(\beta_{I1}\right)\right)}\cdot \left(1-e^{-\left(\frac{a_{P}}{\cos\left(\beta_{I1}\right)}+2a_{P}\right)h}\right)+I_{S}(0)\cdot \frac{s_{P}\left(1-f\right)}{2a_{P}}\cdot \left(1-e^{-4a_{P}h}\right)$$

### 2.4. Equations Describing Light Transmittance and Scattering in the Spongy Mesophyll Layer

Considering the high light scattering coefficient in the spongy mesophyll layer ($s_{Sp}$ = 1000 cm^−1^ [49]), we used the Kubelka–Munk model with four light flows [40,42] to describe optical properties in this layer. We used modified coordinate *x*_1_ (*x*_1_ = *x* − *h*) to simplify the analysis. *x*_1_ can be used from *h* to *h* + l only, where *h* and *l* are the thicknesses of the palisade and spongy mesophyll layers, respectively. System of Equation (39) shows the initial Kubelka–Munk equations:

(39) $$\frac{dI_{C}(x_{1})}{dx_{1}}=-\frac{a_{Sp}+s_{Sp}}{\cos\left(\beta_{I1}\right)}\cdot I_{C}(x_{1})\;\frac{dJ_{C}(x_{1})}{dx_{1}}=\frac{a_{Sp}+s_{Sp}}{\cos\left(\beta_{I2}\right)}\cdot J_{C}(x_{1})\;\begin{array}{l} \frac{dI_{S}(x_{1})}{dx_{1}}=f\cdot \frac{s_{Sp}}{\cos\left(\beta_{I1}\right)}\cdot I_{C}(x_{1})+\left(1-f\right)\cdot \frac{s_{Sp}}{\cos\left(\beta_{I2}\right)}\cdot J_{C}(x_{1})-\\ -2\left(a_{Sp}+s_{Sp}\cdot \left(1-f\right)\right)\cdot I_{S}(x_{1})+2s_{Sp}\cdot \left(1-f\right)\cdot J_{S}(x_{1}) \end{array}\;\begin{array}{l} \frac{dJ_{S}(x_{1})}{dx_{1}}=-\left(1-f\right)\cdot \frac{s_{Sp}}{\cos\left(\beta_{I1}\right)}\cdot I_{C}(x_{1})-f\cdot \frac{s_{Sp}}{\cos\left(\beta_{I2}\right)}\cdot J_{C}(x_{1})-\\ -2s_{Sp}\cdot \left(1-f\right)\cdot I_{S}(x_{1})+2\left(a_{Sp}+s_{Sp}\cdot \left(1-f\right)\right)\cdot J_{S}(x_{1}) \end{array}$$

where $a_{Sp}$ and $s_{Sp}$ are light absorption and scattering coefficients in the spongy mesophyll.

System of Equation (39) was transformed to the system of Equation (40) to simplify analysis:

(40) $$\frac{dI_{C}(x_{1})}{dx_{1}}=L_{11}\cdot I_{C}(x_{1})\;\frac{dJ_{C}(x_{1})}{dx_{1}}=L_{22}\cdot J_{C}(x_{1})\;\frac{dI_{S}(x_{1})}{dx_{1}}=L_{31}\cdot I_{C}(x_{1})+L_{32}\cdot J_{C}(x_{1})+L_{33}\cdot I_{S}(x_{1})+L_{34}\cdot J_{S}(x_{1})\;\frac{dJ_{S}(x_{1})}{dx_{1}}=L_{41}\cdot I_{C}(x_{1})+L_{42}\cdot J_{C}(x_{1})+L_{43}\cdot I_{S}(x_{1})+L_{44}\cdot J_{S}(x_{1})$$

where coefficients (*L*) correspond to coefficients of light flows in the system of Equation (39).

We used the method of undetermined coefficients describing light flows as the combination of elementary exponents ($I_{C}(x)=Ae^{\lambda x_{1}}$, $J_{C}(x)=Be^{\lambda x_{1}}$, $I_{S}(x)=Ce^{\lambda x_{1}}$, and $J_{S}(x)=De^{\lambda x_{1}}$)

Equation (41) is the characteristic equation of this system:

(41) $$\left(L_{11}-\lambda \right)\cdot \left(L_{22}-\lambda \right)\cdot \left[\left(L_{33}-\lambda \right)\cdot \left(L_{44}-\lambda \right)-L_{34}L_{43}\right]=0$$

The solution of this equation is Equation (42):

(42) $$\lambda_{1}=L_{11}\;\lambda_{2}=L_{22}\;\lambda_{3}=\frac{L_{33}+L_{44}}{2}+\sqrt{{\left(\frac{L_{33}+L_{44}}{2}\right)}^{2}-\left(L_{33}\cdot L_{44}-L_{34}\cdot L_{43}\right)}\;\lambda_{4}=\frac{L_{33}+L_{44}}{2}-\sqrt{{\left(\frac{L_{33}+L_{44}}{2}\right)}^{2}-\left(L_{33}\cdot L_{44}-L_{34}\cdot L_{43}\right)}$$

Thus, the system of Equation (43) was used to describe the optical properties of the spongy mesophyll layer (for *x* ranging from *h* to *h* + *l*):

(43) $$I_{C}(x)=A_{1}e^{\lambda_{1}\left(x-h\right)}+A_{2}e^{\lambda_{2}\left(x-h\right)}+A_{3}e^{\lambda_{3}\left(x-h\right)}+A_{4}e^{\lambda_{4}\left(x-h\right)}\;J_{C}(x)=B_{1}e^{\lambda_{1}\left(x-h\right)}+B_{2}e^{\lambda_{2}\left(x-h\right)}+B_{3}e^{\lambda_{3}\left(x-h\right)}+B_{4}e^{\lambda_{4}\left(x-h\right)}\;I_{S}(x)=C_{1}e^{\lambda_{1}\left(x-h\right)}+C_{2}e^{\lambda_{2}\left(x-h\right)}+C_{3}e^{\lambda_{3}\left(x-h\right)}+C_{4}e^{\lambda_{4}\left(x-h\right)}\;J_{S}(x)=D_{1}e^{\lambda_{1}\left(x-h\right)}+D_{2}e^{\lambda_{2}\left(x-h\right)}+D_{3}e^{\lambda_{3}\left(x-h\right)}+D_{4}e^{\lambda_{4}\left(x-h\right)}$$

where *A*_1_, *A*_2_, *A*_3_, *A*_4_, *B*_1_, *B*_2_, *B*_3_, *B*_4_, *C*_1_, *C*_2_, *C*_3_, *C*_4_, *D*_1_, *D*_2_, *D*_3_, and *D*_4_ are constants.

System of Equation (44) was used for the description of boundary conditions:

(44) $$A_{1}+A_{2}+A_{3}+A_{4}=I_{C}(h)\;B_{1}e^{\lambda_{1}l}+B_{2}e^{\lambda_{2}l}+B_{3}e^{\lambda_{3}l}+B_{4}e^{\lambda_{4}l}=J_{C}(h+l)\;C_{1}+C_{2}+C_{3}+C_{4}=I_{S}(h)\;D_{1}e^{\lambda_{1}l}+D_{2}e^{\lambda_{2}l}+D_{3}e^{\lambda_{3}l}+D_{4}e^{\lambda_{4}l}=J_{S}(h+l)$$

*I_C_*(*x*) and *J_C_*(*x*) cannot be dependent on other light flows. It means that *A*_2_ = *A*_3_ = *A*_4_ = 0 and *B*_1_ = *B*_3_ = *B*_4_ = 0; in contrast, *A*_1_ = *I_C_*(*h*) and *B*_2_ = *J_C_*(*h* + *l*).

Based on elementary exponents and Equation (40) for *I_S_*(*x*) and *J_S_*(*x*), the system of Equation (45) was derived:

(45) $$C\cdot \left(L_{33}-\lambda \right)+D\cdot L_{34}=-\left(A\cdot L_{31}+B\cdot L_{32}\right)\;C\cdot L_{43}+D\cdot \left(L_{44}-\lambda \right)=-\left(A\cdot L_{41}+B\cdot L_{42}\right)$$

Equations (46) and (47) are solutions of this system at *λ*_1_ (*A*_1_ = *I_C_*(*h*) and *B*_1_ = 0):

(46) $$C_{1}=I_{C}(h)\cdot \frac{-L_{31}\cdot \left(L_{44}-\lambda_{1}\right)+L_{41}\cdot L_{34}}{\left(L_{33}-\lambda_{1}\right)\cdot \left(L_{44}-\lambda_{1}\right)-L_{34}\cdot L_{43}}$$

(47) $$D_{1}=I_{C}(h)\cdot \frac{-L_{41}\cdot \left(L_{33}-\lambda_{1}\right)+L_{31}\cdot L_{43}}{\left(L_{33}-\lambda_{1}\right)\cdot \left(L_{44}-\lambda_{1}\right)-L_{34}\cdot L_{43}}$$

Equations (48) and (49) are solutions of this system at *λ*_2_ (*A*_2_ = 0 and $B_{2}=J_{C}(h+l)\cdot e^{-\lambda_{2}l}$):

(48) $$C_{2}=J_{C}(h+l)\cdot e^{-\lambda_{2}l}\cdot \frac{-L_{32}\cdot \left(L_{44}-\lambda_{2}\right)+L_{42}\cdot L_{34}}{\left(L_{33}-\lambda_{2}\right)\cdot \left(L_{44}-\lambda_{2}\right)-L_{34}\cdot L_{43}}$$

(49) $$D_{2}=J_{C}(h+l)\cdot e^{-\lambda_{2}l}\cdot \frac{-L_{42}\cdot \left(L_{33}-\lambda_{2}\right)+L_{32}\cdot L_{43}}{\left(L_{33}-\lambda_{2}\right)\cdot \left(L_{44}-\lambda_{2}\right)-L_{34}\cdot L_{43}}$$

Equations (50) and (51) describe relations between *C*_3_ and *D*_3_ for *λ*_3_ and *C*_4_ and *D*_4_ for *λ*_4_:

(50) $$D_{3}=-C_{3}\cdot \frac{L_{33}-\lambda_{3}}{L_{34}}$$

(51) $$D_{4}=-C_{4}\cdot \frac{L_{33}-\lambda_{4}}{L_{34}}$$

Based on the system of Equation (44), the system of Equation (52) was derived:

(52) $$C_{3}+C_{4}=I_{S}(h)-C_{1}-C_{2}\;D_{3}e^{\lambda_{3}l}+D_{4}e^{\lambda_{4}l}=J_{S}(h+l)-D_{1}e^{\lambda_{1}l}-D_{2}e^{\lambda_{2}l}$$

where *C*_1_, *C*_2_, *D*_1_, and *D*_2_ can be calculated with Equations (46)–(49). Combining Equations (50)–(52), we derived the system of Equation (53):

(53) $$C_{3}+C_{4}=I_{S}(h)-C_{1}-C_{2}\;C_{3}\frac{L_{33}-\lambda_{3}}{L_{34}}e^{\lambda_{3}l}+C_{4}\frac{L_{33}-\lambda_{4}}{L_{34}}e^{\lambda_{4}l}=-J_{S}(h+l)+D_{1}e^{\lambda_{1}l}+D_{2}e^{\lambda_{2}l}$$

Equations (54) and (55) are solutions of this system:

(54) $$C_{3}=\frac{\left(I_{S}(h)-C_{1}-C_{2}\right)\cdot \left(L_{33}-\lambda_{4}\right)e^{\lambda_{4}l}-\left(-J_{S}(h+l)+D_{1}e^{\lambda_{1}l}+D_{2}e^{\lambda_{2}l}\right)\cdot L_{34}}{\left(L_{33}-\lambda_{4}\right)e^{\lambda_{4}l}-\left(L_{33}-\lambda_{3}\right)e^{\lambda_{3}l}}$$

(55) $$C_{4}=\frac{\left(-J_{S}(h+l)+D_{1}e^{\lambda_{1}l}+D_{2}e^{\lambda_{2}l}\right)\cdot L_{34}-\left(I_{S}(h)-C_{1}-C_{2}\right)\cdot \left(L_{33}-\lambda_{3}\right)e^{\lambda_{3}l}}{\left(L_{33}-\lambda_{4}\right)e^{\lambda_{4}l}-\left(L_{33}-\lambda_{3}\right)e^{\lambda_{3}l}}$$

Combining Equations (50), (51), (54) and (55), we derived Equations (56) and (57) to calculate *D*_3_ and *D*_4_:

(56) $$D_{3}=-\frac{L_{33}-\lambda_{3}}{L_{34}}\frac{\left(I_{S}(h)-C_{1}-C_{2}\right)\cdot \left(L_{33}-\lambda_{4}\right)e^{\lambda_{4}l}-\left(-J_{S}(h+l)+D_{1}e^{\lambda_{1}l}+D_{2}e^{\lambda_{2}l}\right)\cdot L_{34}}{\left(L_{33}-\lambda_{4}\right)e^{\lambda_{4}l}-\left(L_{33}-\lambda_{3}\right)e^{\lambda_{3}l}}$$

(57) $$D_{4}=-\frac{L_{33}-\lambda_{4}}{L_{34}}\frac{\left(-J_{S}(h+l)+D_{1}e^{\lambda_{1}l}+D_{2}e^{\lambda_{2}l}\right)\cdot L_{34}-\left(I_{S}(h)-C_{1}-C_{2}\right)\cdot \left(L_{33}-\lambda_{3}\right)e^{\lambda_{3}l}}{\left(L_{33}-\lambda_{4}\right)e^{\lambda_{4}l}-\left(L_{33}-\lambda_{3}\right)e^{\lambda_{3}l}}$$

### 2.5. Description of Several Iterations of the Light Propagation Through Leaf

Equations from Section 2.2, Section 2.3 and Section 2.4 can be used for the first iteration of the calculation of light transmittance and reflectance. However, Equations (13), (14), (25) and (26) show that light can secondarily input into the leaf lamina; that is, the second iteration of calculation of the light propagation is possible (as well as the third iteration, fourth iterations, fifth iteration, etc.). This effect can be large at low light absorption coefficients (particularly for the near-infrared light, NIR [3]).

The second iteration of calculation of the light propagation through the leaf and light transmittance from lamina to air was based on the same equations from Section 2.2, Section 2.3 and Section 2.4, which were used for the first iteration, after substitution of the following parameters:$I_{C}(0)={I_{C}}^{RI}$, $J_{C}(h+l)={J_{C}}^{RI}$, $I_{S}(0)={I_{S}}^{RI}$, $J_{S}(h+l)={J_{S}}^{RI}$, $\beta_{I1}=\beta_{I2}$, and $\beta_{I2}=\beta_{I1}$.

Calculated after this procedure *I_C_^RI^*, *J_C_^RI^*, *I_S_^RI^*, and *J_S_^RI^* could be used for the third iteration of calculation, etc. Thus, Equations (58) and (59) could be used for the calculation of light outputs from adaxial and abaxial leaf surfaces (*J_out_* and *I_out_*, respectively):

(58) $$J_{out}=\sum_{i=1}^{N}{J_{out}}^{i}$$

(59) $$I_{out}=\sum_{i=1}^{N}{I_{out}}^{i}$$

where *J_out_^i^* and *I_out_^i^* are light outputs from adaxial and abaxial leaf surfaces, respectively, which are calculated on iteration *i*, and *N* is the quantity of the iterations, which is necessary to approximately describe the reflectance and transmittance spectra of leaves.

### 2.6. Description of Light Absorption Coefficients

In the current model, we considered that the light absorption coefficient was the function of the light wavelength through photosynthetic pigments and complexes formed by these pigments [49,56,57,58]. Maier et al. [49] assumed that the concentration of photosynthetic pigments in the spongy mesophyll layer was 20% of this concentration in the palisade mesophyll layer (*N*_*Sp*/*P*_ = 0.2):

(60) $$a_{Sp}\left(\lambda \right)=N_{Sp/P}\cdot a_{P}\left(\lambda \right)$$

where $a_{P}\left(\lambda \right)$ and $a_{Sp}\left(\lambda \right)$ are spectra of the light absorption coefficient of layers of the palisade and spongy mesophyll, respectively. $a_{P}\left(\lambda \right)$ and $a_{Sp}\left(\lambda \right)$ were used as $a_{P}$ and $a_{Sp}$ in the analysis.

**Figure 3:**
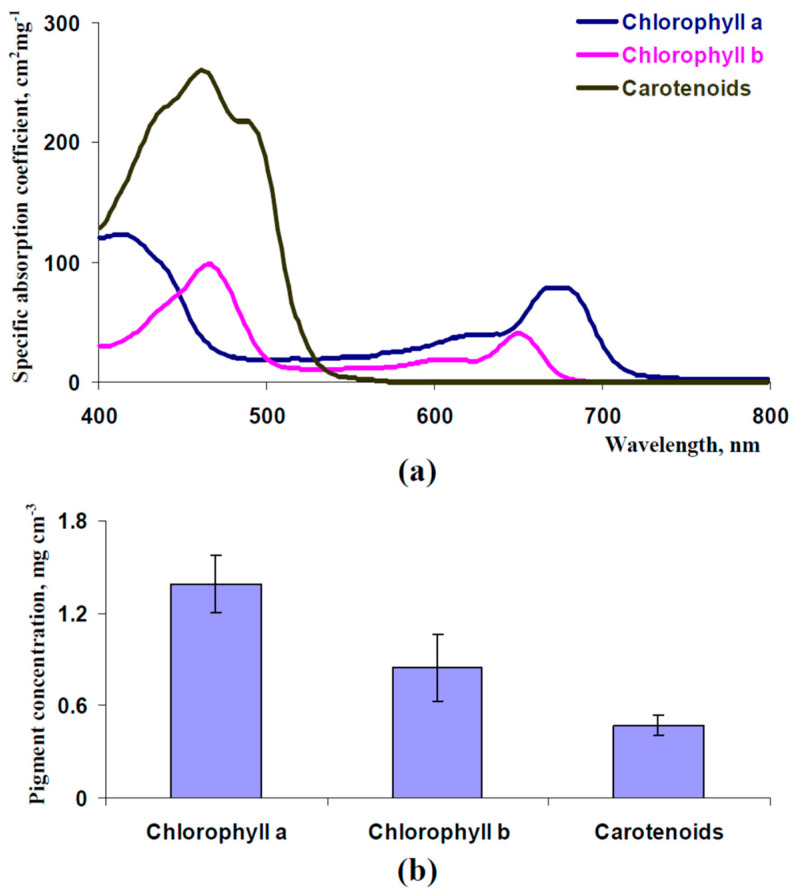
Spectra of specific light absorption coefficients for chlorophyll a, chlorophyll b, and carotenoids (on an example of β-carotene) (**a**) and average concentrations of these pigments in pea leaves (*n* = 6) (**b**). The spectra of light absorption were constructed on the basis of [58]. The pigment concentrations were experimentally measured. The second mature leaf was used. Pea plants were cultivated for 16 days under moderate light intensity.

Equation (61) was used for the calculation of $a_{P}\left(\lambda \right)$:

(61) $$a_{P}\left(\lambda \right)=C_{ChA}\cdot a_{ChA}\left(\lambda \right)+C_{ChB}\cdot a_{ChB}\left(\lambda \right)+C_{Car}\cdot a_{Car}\left(\lambda \right)$$

where *C_ChA_*, *C_ChB_*, and *C_Car_* are concentrations (mg cm^−3^) of chlorophyll a, chlorophyll b, and carotenoids, respectively, in the palisade mesophyll, and $a_{ChA}\left(\lambda \right)$, $a_{ChB}\left(\lambda \right)$, and $a_{Car}\left(\lambda \right)$ are spectra of specific light absorption coefficients (cm^2^ mg^−1^) of chlorophyll a, chlorophyll b, and carotenoids, respectively. These spectra were constructed on the basis of [58] (Figure 3a).

Equation (62) was used for the calculation of the average concentration of each photosynthetic pigment (*C^av^*) in leaves to compare with experimental concentrations (Figure 3b):

(62) $$C^{av}=C\cdot \left(\frac{h}{l+h}+N_{Sp/P}\cdot \frac{l}{l+h}\right)$$

where *C* is the pigment concentration in the palisade mesophyll layer.

### 2.7. Experimental Methods Used to Parameterization and Verification of the Model

Peas (*Pisum sativum* L., variety “Albumen”), which are dicot plants, were used to parameterize and verify the developed model of leaf reflectance. Plants were cultivated in pots with universal soil (nine plants per pot) under conditions of a vegetation room (16 h photoperiod and 24 °C); plants were irrigated three times a week. Luminescent lamps FSL YZ18RR (Foshan Electrical And Lighting Co., Ltd., Foshan, China) were used for the illumination. There were two variants of the illumination intensity (Figure S1) as follows: moderate (about 55 µmol m^−2^s^−1^) and low (about 15 µmol m^−2^s^−1^); these intensities were controlled using the Thorlabs PM100D optical power meter with an S120VC sensor (200–1100 nm) (Thorlabs Inc., Newton, MA, USA). Here, 9-day-old and 16-day-old pea plants were investigated in different variants of the experiment. Moreover, 16-day-old pea plants, which were cultivated under the 55 µmol m^−2^s^−1^ light intensity, were used to parameterize the model. All experimental variants were used to verify the developed model.

Measuring leaf reflectance spectra, content of chlorophylls and carotenoids, and thickness of the palisade and spongy mesophyll layers were analyzed. Second mature leaves were investigated. All noted measurements were carried out on the same leaves.

Reflectance spectra were measured in attached pea leaves before other measurements. The handheld PolyPen RP 410 UVIS system (Photon Systems Instruments, Drásov, Czech Republic), which is the specialized system to measure spectral characteristics of leaf reflectance in plants, was used.

Cross-sections of pea leaves were manually prepared using a razor blade in accordance with the widely used botanical method. The leaf fragment was fixed by two plates of foam plastic; further, cross-sections were cut, placed into a water drop on the microscope slide, and fixed by the cover glass. Microscope MT 4200 with lens Plan Achromat 40X and ocular micrometer (Meiji Techno, Saitama, Japan) was used to estimate thicknesses of the palisade and spongy mesophyll layers.

Concentrations of chlorophyll a, chlorophyll b, and carotenoids were measured using the standard biochemical method [59,60,61] of spectrophotometry of acetone extract from pea leaves using an SF-2000 UV/Vis Spectrophotometer (OKB Spectr, St. Petersburg, Russia). Optical densities were measured at 440.5, 644, and 662 nm; concentrations of photosynthetic pigments were calculated on the basis of Holm–Wettstein equations.

## 3. Results

### 3.1. Parameterization of the Developed Leaf Reflectance Model

Parameterization of the developed model of leaf reflectance was the first task. Thicknesses of palisade and spongy mesophyll layers (Figure 2b) and concentrations of the chlorophyll a, chlorophyll b, and carotenoids (Figure 3b) were estimated using experimental methods in the leaves of pea plants (see Section 2.7). Measured reflectance spectra of these leaves were used to check the accuracy of the developed model. It should be noted that pea leaves were used as an example of leaves of dicot plants, which have palisade and spongy mesophyll layers [38]; the model cannot be used to describe reflectance of leaves of monocot plants with uniform mesophyll.

The following parameters were used for initial parameterization. Average experimental values of thicknesses (*h* = 35.5 μm and *l* = 58.6 μm) were used. C_ChA_ = 2.77 mg cm^−3^, C_ChB_ = 1.69 mg cm^−3^, and C_Car_ = 0.94 mg cm^−3^ were used because average concentrations of chlorophyll a, chlorophyll b, and carotenoids in leaves, which were calculated on basis of these C_ChA_, C_ChB_, and C_Car_, Equation (62), and *N_Sp_*_/*P*_ = 0.2 (47), corresponded to experimental ones. $a_{ChA}\left(\lambda \right)$, $a_{ChB}\left(\lambda \right)$, and $a_{Car}\left(\lambda \right)$ were constructed on the basis of [58] (Figure 3a). Values of other parameters were *β_O_*_1_ = 35º (in accordance with the angle of the leaf illumination performed using PolyPen RP 410), *I*_0_ = 1000 μmol m^−2^s^−1^ and *J*_0_ = 0 μmol m^−2^s^−1^ (assumed), *F_S_* = 0 (assumed), *n_I_* = 1.415 [48], *f* = 0.5 (assumed), =5 cm^−1^, and =1000 cm^−1^ [47].

**Figure 4:**
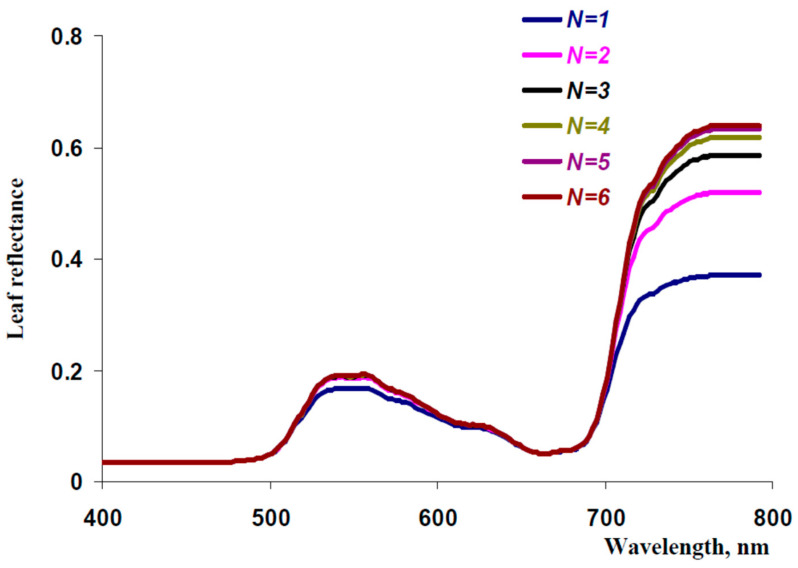
Spectra of leaf reflectance calculated on basis of the developed model with different quantities of the iterations (*N*). The leaf reflectance was calculated as a ratio of the sum of all components of backward collimated and backward scattered light transferring through the adaxial leaf surface (*J_out_*) to the intensity of incident light (*I*_0_). The influence of *N* on *J_out_* was calculated in accordance with Equation (58). The following parameters were used (see Section 2.1 for details): *β*_O1_ = 35° (in accordance with angle of the leaf illumination by PolyPen RP 410), *I_0_* = 1000 μmol m^−2^s^−1^ and *J*_0_ = 0 μmol m^−2^s^−1^ (assumed), *F_S_* = 0 (assumed), *n_I_* = 1.415 [50], *f* = 0.5 (assumed), *h* = 35.5 μm and *l* = 58.6 μm (Figure 2b), $s_{P}=$ 5 cm^−1^ and $s_{Sp}$ = 1000 cm^−1^ [49], C_ChA_ = 2.77 mg cm^−3^, C_ChB_ = 1.69 mg cm^−3^, and C_Car_ = 0.94 mg cm^−3^ corresponded to average experimental concentrations of these pigments (1.39, 0.85, and 0.47 mg cm^−3^, respectively, Figure 3b) at *N_Sp/P_* = 0.2 [49]); $a_{ChA}\left(\lambda \right)$, $a_{ChB}\left(\lambda \right)$, and $a_{Car}\left(\lambda \right)$ are shown in Figure 3a.

Figure 4 shows model-based reflectance spectra, which were calculated at different quantities of the iterations (*N*) in accordance with Equation (58). It was shown that reflectance of visible light was accurately described at *N* = 2 (or more) because increasing *N* did not influence the reflectance spectra at *N* ≥ 2. Moreover, *N* = 1 could be used for the calculation of the leaf reflectance in blue and red spectral regions; a small error was observed in the green spectral region only. In contrast, an accurate description of the NIR reflectance was observed at *N* ≥ 5 because increasing *N* influenced model-based leaf reflectance spectra at *N* ≤ 5. As a result, we used *N* = 6 for further analysis to completely exclude technical error, which could disrupt results at low *N*.

**Figure 5:**
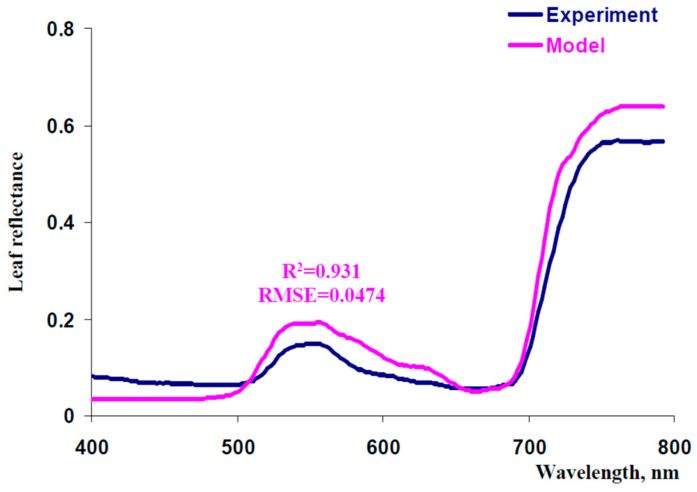
Experimental and model-based spectra of leaf reflectance. The average experimental reflectance spectrum (*n* = 6) measured in the second mature leaf of pea plants (PolyPen RP 410) is shown. Standard errors are not shown because they are small. Pea plants were cultivated for 16 days under the moderate light intensity. Parameters of the model are shown in Figure 4; *N* = 6 was used for the analysis. R^2^ and RMSE are the determination coefficient and root mean square error between the experimental and model-based spectra.

Figure 5 shows experimental and model-based spectra of leaf reflectance. The model-based spectrum was calculated at initial values of parameters (see above) and *N* = 6. This result showed that the model-based spectrum was similar to the experimental one in a qualitative manner; however, quantity differences were also observed (especially in blue, green, and NIR spectral regions).

**Figure 6:**
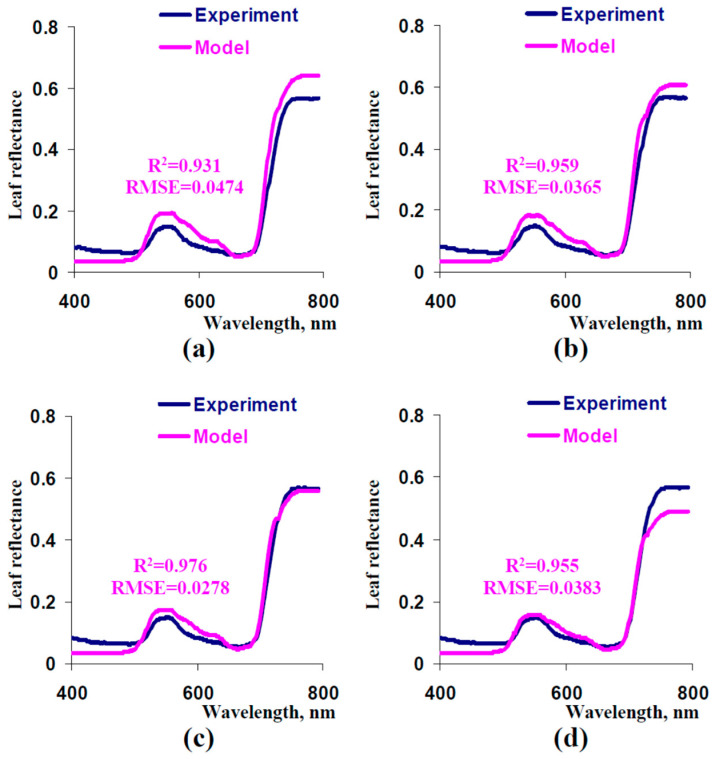
Model-based spectra of leaf reflectance at $s_{Sp}=$ 1000 cm^−1^ (**a**), $s_{Sp}=$ 800 cm^−1^ (**b**), $s_{Sp}=$ 600 cm^−1^ (**c**), and $s_{Sp}=$ 400 cm^−1^ (**d**) and the experimental spectrum (from Figure 5). Other parameters of the model were the same as the parameters that were used for the simulation of the spectrum in Figure 5. R^2^ and RMSE are, respectively, the determination coefficient and root mean square error between the experimental and model-based spectra.

An additional parameterization of the developed model was the next task. First, we analyzed the influence of $s_{Sp}$ on the accuracy of description of experimental reflectance spectra (Figure 6). It was shown that decreasing $s_{Sp}$ from 1000 cm^−1^ to 600 cm^−1^ increased this accuracy (Figure 6a–c); in contrast, decreasing s_Sp_ from 600 cm^−1^ to 400 cm^−1^ decreased similarity between model-based and experimental spectra (Figure 6c). As a result, we used =600 cm^−1^ in further analysis.

**Figure 7:**
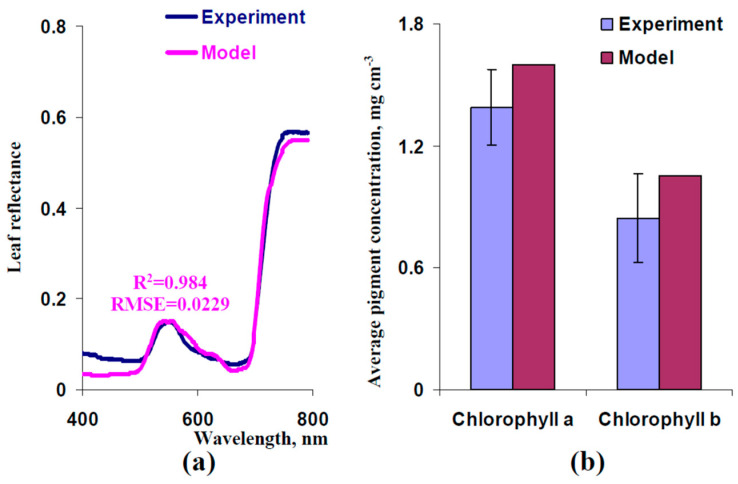
(**a**) Model-based spectrum of leaf reflectance at C_ChA_ = 3.19 mg cm^−3^ and C_ChB_ = 2.09 mg cm^−3^ and the experimental spectrum (from Figure 5). Other parameters of the model were the same as the parameters that were used for the simulation of the spectrum in Figure 6c. R^2^ and RMSE are, respectively, the determination coefficient and root mean square error between the experimental and model-based spectra. (**b**) Average concentrations of chlorophyll a and b, which corresponded to C_ChA_ = 3.19 mg cm^−3^ and C_ChB_ = 2.09 mg cm^−3^, and their experimental concentrations in pea leaves (form Figure 3).

Second, we used small corrections of C_ChA_ and C_ChB_ to increase the determination coefficient between experimental and model-based reflectance spectra. It was shown that using C_ChA_ = 3.19 mg cm^−3^ and C_ChB_ = 2.09 mg cm^−3^ improved the accuracy of the description of the experimental leaf reflectance spectrum (Figure 7a). Average leaf concentrations of chlorophyll a and b, which were calculated on the basis of these values of C_ChA_ and C_ChB_, were similar to experimental ones (differences approximately equaled to standard errors of experimental values of concentrations of chlorophylls). Thus, we used corrected C_ChA_ and C_ChB_ in further analysis of the model.

**Figure 8:**
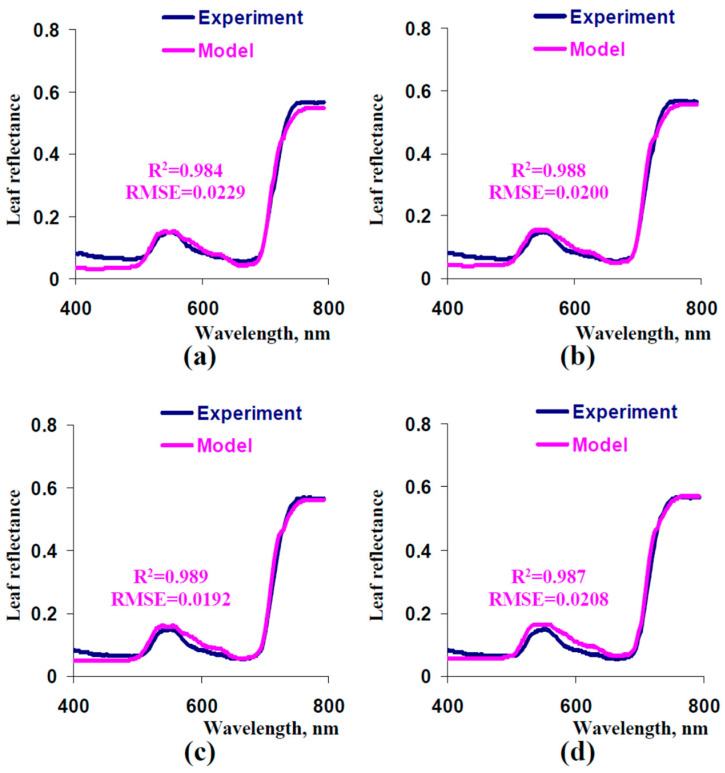
Model-based spectra of leaf reflectance at *F_S_* = 0 (**a**), *F_S_* = 0.075 (**b**), *F_S_* = 0.15 (**c**), and *F_S_* = 0.225 (**d**) and the experimental spectrum (from Figure 5). Other parameters of the model were the same as the parameters that were used for the simulation of the spectrum in Figure 7. R^2^ and RMSE are, respectively, the determination coefficient and root mean square error between the experimental and model-based spectra.

Third, we analyzed the influence of the fraction of the rough surface (*F_S_*) on reflectance spectra to decrease the error of the model-based leaf reflectance spectra in the blue spectral region. It was shown (Figure 8) that increasing *F_S_* to 0.1–0.225 improved description of the experimental leaf reflectance spectrum (especially in the blue spectral region). As a result, we used *F_S_* = 0.15 in the further analysis of the model.

Thus, additional parameterization showed that relatively minor changes in parameters provided increasing the determination coefficient between experimental and model-based leaf reflectance spectra from about 0.93 to about 0.99. This improvement was especially large in blue, green, and NIR spectral regions.

### 3.2. Verification and Analysis of the Developed Leaf Reflectance Model

The next task of the investigation was the verification of the developed model on the basis of experimental leaf reflectance spectra. We used plants, which were cultivated under moderate (about 55 µmol m^−2^s^−1^) and low (about 15 µmol m^−2^s^−1^) light intensities, to provide varying leaf characteristics because light intensity is an important factor influencing leaf anatomy and the content of photosynthetic pigments [62,63,64]. Two ages of pea plants (9 and 16 days) were used to additional check results.

**Figure 9:**
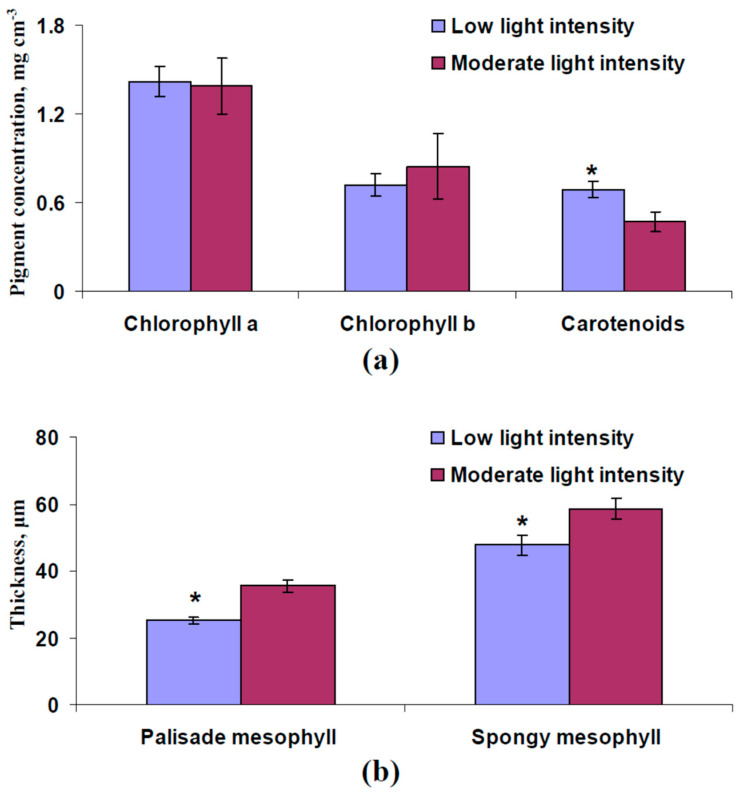
Experimental concentrations of chlorophyll a, chlorophyll b, and carotenoids (**a**) and thicknesses of palisade and spongy mesophyll (**b**) in second mature leaves of pea plants, which were cultivated for 16 days under low and moderate light intensity (*n* = 6). *, the value is significantly different from this value in plants cultivated under moderate light intensity.

**Figure 10:**
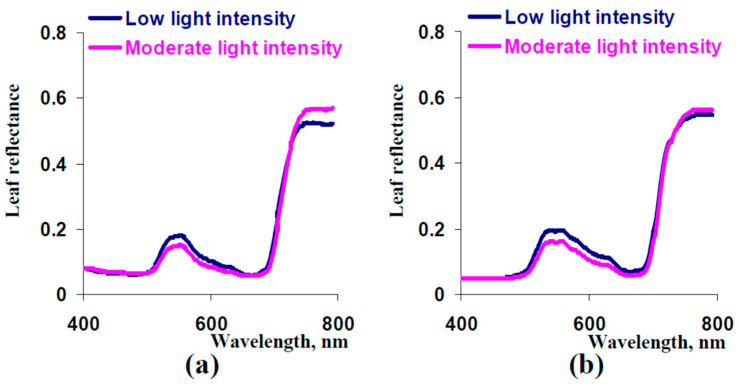
Experimental (**a**) and model-based (**b**) spectra of leaf reflectance in pea plants, which were cultivated for 16 days under low and moderate light intensity (*n* = 6 for experiments). The average experimental reflectance spectrum (*n* = 6) measured in the second mature leaf of pea plants (PolyPen RP 410) is shown. Standard errors are not shown because they are small. In variant “Moderate light intensity”, parameters of the model were the same as the parameters that were used for the simulation of the spectrum in Figure 7. In variant “Low light intensity”, *h* = 25.2 μm and *l* = 47.8 μm (see Figure 9b); other parameters were not changed. R^2^ between experimental and model-based dependences were 0.989 (variant “moderate light intensity”) and 0.982 (variant “low light intensity”).

It was shown (Figure 9) that cultivation under low light intensity significantly decreased thicknesses of palisade and spongy mesophyll layers and increased the content of carotenoids in 16-day-old pea plants. The concentrations of chlorophylls were not dependent on the light intensity during cultivation. Figure 10 shows experimental and model-based leaf reflectance spectra. It was experimentally shown that cultivation of pea plants under low light intensity increased reflectance in the green spectral range and decreased reflectance in the NIR spectral range (Figure 10a). Analysis of the developed model showed similar results as follows: low thicknesses of palisade and spongy mesophyll layers (equaling to experimental thicknesses in plants cultivated under low light intensity) increased reflectance in the green spectral range and decreased reflectance in the NIR spectral range (Figure 10b). In contrast, changes in the carotenoid concentration weakly influenced the model-based leaf reflectance spectrum (Figure S2). The last result showed that the difference in the carotenoid concentration was not a probable reason for changes in the leaf reflectance spectrum in pea plants cultivated under low light intensity.

A similar experimental investigation of 9-day-old pea plants showed (Figure S3) that cultivation under the low light intensity did not influence concentrations of chlorophylls and carotenoids; in contrast, thicknesses of palisade and spongy mesophyll layers were decreased in this case. Decreased thicknesses of palisade and spongy mesophyll layers were accompanied by increasing reflectance in the green spectral range and decreasing reflectance in the NIR spectral range in both experimental and model-based reflectance spectra (Figure S4). This result additionally supported the key role of changes in thicknesses in changes of reflectance because the carotenoid concentration was not influenced by cultivation under the low light intensity in the last experiment.

Thus, it was shown that the developed model could simulate the dependence of the leaf reflectance spectrum on thicknesses of palisade and spongy mesophyll layers. The similarity of changes was observed for two experimental groups (9-day-old and 16-day-old pea plants). This result verified the developed leaf reflectance model and showed the possibility of using the model to analyze factors influencing reflectance.

**Figure 11:**
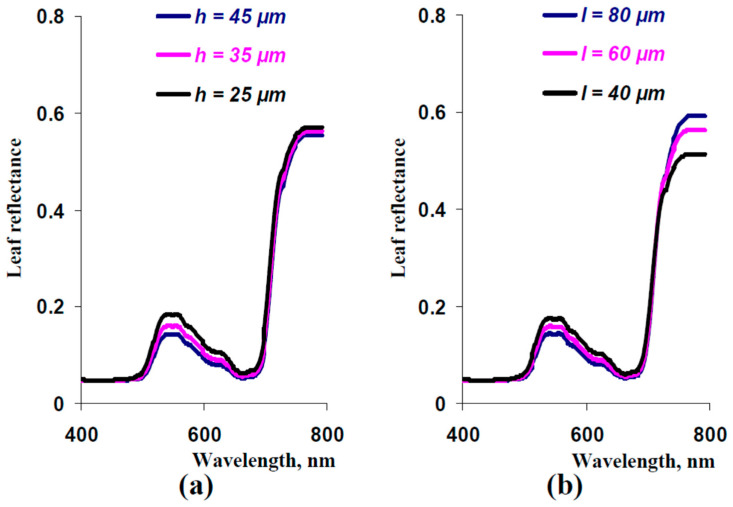
Spectra of leaf reflectance calculated at *h* = 45 μm, *h* = 35 μm, and *h* = 25 μm (**a**) and at *l* = 80 μm, *l* = 60 μm, and *l* = 40 μm (**b**). Other parameters of the model were the same as the parameters that were used for the simulation of the spectrum in Figure 8c.

It should be finally noted that changes in the model-based leaf reflectance spectrum were shown at simultaneous decreasing thicknesses of palisade and spongy mesophyll layers (in accordance with the experimental decreasing). Next, we used the developed model to analyze the influence of changes in thickness of the palisade mesophyll layer only and in thickness in the spongy mesophyll layer only. It was shown (Figure 11a) that decreasing the thickness of the palisade mesophyll layer increased reflectance in the green spectral region but did not influence reflectance in the NIR spectral region. In contrast, decreasing the thickness of the sponge mesophyll layer both increased reflectance in the green spectral region and decreased reflectance in the NIR spectral region (Figure 11b).

Thus, the last results of the analysis of the developed model showed that decreasing thicknesses of both palisade and spongy mesophyll layers stimulated reflectance in the green spectral region. In contrast, decreasing the reflectance in the NIR spectral region was caused by decreasing the thickness of the spongy mesophyll layer only.

## 4. Discussion

Multispectral and hyperspectral imaging is an important tool of remote sensing of plants that can be used to estimate their growth and development [12,13,14,15], photosynthesis [16,17,18,19,20], nitrogen content [11,12], water content [8,10], concentrations of pigments [5,6,7], and other characteristics, including revealing changes in these parameters under the action of stressors. Potentially, using reflectance in narrow bands and calculating reflectance indices should increase efficiency of the plant remote sensing [1,3]; however, relationships between reflectance in specific narrow spectral bands (and, therefore, corresponding reflectance indices) and specific plant characteristics can be unstable (e.g., strong variability was shown for relationships of photochemical reflectance index to photosynthetic parameters [22,23]). Possible reasons for the instability include changes in photosynthetic pigment composition, variations in leaf anatomy, and different angles between the direction of the incident light and the leaf surface [3].

The development of mathematical models describing the optical properties of leaves provides theoretical tools to reveal the influence of noted reasons on reflectance spectra and to minimize this influence. The main result of the current study is the development, parameterization, and verification of the analytical model to describe leaf reflectance spectra in dicot plants. This model describes light flows through the leaf surface on the basis of Snell’s and Fresnel’s laws [51,53,65]. Fractions of smooth and rough surfaces, which provide transmittance and reflectance collimated and scattered light [65,66], are also considered in the developed model. Light flows in the palisade mesophyll, which has a low light scattering coefficient [49], are mainly described on the basis of the Beer–Bouguer–Lambert law. Light flows in the sponge mesophyll, which has a high light scattering [49], are described on basis of the Kubelka–Munk theory (in accordance with [40,42]). Parameterization and verification of the developed model (with using pea leaves) show that this model accurately describes leaf reflectance spectra (see Figure 8c, Figure 10 and Figure S4), that is, it can be used to analyze data of plant remote sensing on the basis of multispectral and hyperspectral imaging.

The developed model does not require to assume that leaves are described as series of plates; this assumption is the basis of numerous plant optical models, including different variants of PROSPECT and FLUOSPECT [38,66,67,68]. Our model does not require a complex description of light trajectories, which is typically for ray tracing models [38,55,69]. Moreover, combining different approaches (particularly the Beer–Bouguer–Lambert law for the palisade mesophyll and the Kubelka–Munk theory for the spongy mesophyll), the developed model provides a relatively simple description of the optical properties of leaf; the proposed equations have the analytical solution. It should be additionally noted that the developed model can be easily adapted to describe reflectance in monocot plants with one type of leaf mesophyll (*h* = 0 µm and *N_Sp/P_* = 1 should be used).

The additional result of the current study is the theoretical revealing of factors that influence the leaf reflectance in different spectral ranges. Particularly, reflectance in the NIR spectral range can be stimulated by increasing the light scattering in the spongy mesophyll (Figure 6) and thickness of this mesophyll (Figure 10 and Figure S4). These effects are in good accordance [29] with the positive influence of the ratio of mesophyll cell surface to intercellular air spaces on reflectance in the NIR spectral region because increasing this ratio should stimulate light scattering in the spongy mesophyll layer; increasing the spongy mesophyll thickness can also increase the NIR reflectance. Influence of the light scattering and thickness of the spongy mesophyll layer can cause variability of vegetation reflectance indices, which use reflectance in the NIR spectral range, including the normalized difference vegetation index [70], optimized soil-adjusted vegetation index [71], green normalized difference vegetation index [72], triangular vegetation index [73], and others.

Reflectance in the green spectral range can be mainly stimulated by increasing the light scattering in the spongy mesophyll (Figure 6) and decreasing thicknesses of both the palisade and spongy mesophyll layers (Figure 10 and Figure S4). These effects can increase the variability of indices that use the green spectral bands, including the photochemical reflectance index [16,17,18,19,20] and its modifications [74,75,76]. It is probable that the increased variability of indices can explain relatively weak relationships between the photochemical reflectance index and photosynthetic parameters [22,23].

Finally, reflectance in the blue spectral region is relatively stable; however, increasing the fraction of the rough surface stimulates reflectance in this region (Figure 8). Considering this effect, it can be expected that reflectance indices, which are used reflectance in the blue spectral region (e.g., normalized difference pigment index [77], normalized phaeophytinization index [78], Carter index 1 [79], and others), are dependent on ratio between areas with smooth and rough leaf surfaces. It should be additionally noted that the leaf surface reflectance is mainly considered as the Fresnel’s reflectance [65]; however, results of parameterization of our model (on basis of pea leaves) show that a fraction of the rough leaf surface can be about 15% and more (Figure 8c,d).

Thus, the developed model of leaf reflectance in dicot plants shows some potential reasons for the variability of reflectance indices and, therefore, the variability of their relationships to plant characteristics. In future investigations, the model can be used both for the complex analysis of the leaf reflectance spectra and for the revealing of new reflectance indices, which will be stably related to plant characteristics.

It should be finally noted that the current model has some assumptions and restrictions that should be considered at its use. (i) The model described two layers of mesophyll (palisade and spongy), which are typical for dicot plants [38]. It means that equations of the model can be potentially used to analyze reflectance of leaves of different dicot plants; however, the model parameterization is mainly based on experimental results (contents of photosynthetic pigments, thicknesses of layers of palisade and spongy mesophyll, and leaf reflectance spectra) shown on pea plants. Thus, quantity analysis of the leaf spectra in other dicot plants can require additional parameterization of the developed model.

(ii) The model does not consider participation of the epidermal layer in the light reflectance and transmittance because this layer is narrow and transparent (see, e.g., Figure 2a); it can be expected that it weakly influences light propagation. However, epidermal cells can also influence leaf optical properties because they focus light on mesophyll cells and, probably, induce additional light scattering [80,81]. The description of the effect is one of the potential ways of model development in the future.

(iii) It is known that the low light intensity is accompanied by stomatal closure [82] and chlorophyll content changes [83,84] modifying photosynthetic processes. Other environmental factors, for example, temperature or humidity, can also influence stomata opening, content of photosynthetic pigments, and photosynthetic parameters [82,85,86]. The current model is focused on the leaf optical properties and does not describe these physiological processes (Figure 1). However, considering the relationship of plant reflectance spectra to concentrations of photosynthetic pigments [3,4,7], photosynthetic activity [17,19,22,23], and water content [8,9], it is probable that description of changes in concentrations of pigments, photosynthesis, and transpiration under action of environmental factors can be integrated to the leaf reflectance model in the future; this “extended” model can provide an effective tool for analysis of results of the plant remote sensing in the future.

## 5. Conclusions

Improving methods of plant remote sensing on the basis of multispectral and hyperspectral imaging requires the development of mathematical models of reflectance in plants. In the current study, we developed the analytical model of the leaf reflectance in dicot plants (on example of pea leaves), which was based on the Snell’s and Fresnel’s laws to describe light reflectance and transmittance on borders of lamina, on the Beer–Bouguer–Lambert law to describe the light transmittance in the palisade mesophyll, and on the Kubelka–Munk theory to describe light transmittance and scattering in the spongy mesophyll. The model was parameterized and verified using the experimental results and the literature data. Analysis of the model theoretically showed some factors influencing the leaf reflectance spectra (e.g., the coefficient of light scattering in the spongy mesophyll, thicknesses of both mesophyll layers, fraction of the rough surface in the leaf, and others).

In future investigations, the developed model can be used both for the complex analysis of leaf reflectance spectra to improve interpretation of results of remote sensing and for revealing new reflectance indices, which will be stably related to plant characteristics.

## Data Availability

The original contributions presented in this study are included in the article/Supplementary Materials; further inquiries can be directed to the corresponding author.

## Supplementary Materials

The following supporting information can be downloaded at: https://www.mdpi.com/article/10.3390/plants13223258/s1, Figure S1: Scheme of cultivation of pea and measurements of its parameters (a) and photos of plants, which were cultivated under moderate and low light intensity (b); Figure S2: Spectra of leaf reflectance calculated at *C_Car_* = 1.5 mg cm^−3^, *C_Car_* = 1 mg cm^−3^, and *C_Car_* = 0.5 mg cm^−3^; Figure S3: Experimental concentrations of chlorophyll a, chlorophyll b, and carotenoids (a) and thicknesses of palisade and spongy mesophyll (b) in second mature leaves of pea plants, which were cultivated for 9 days under the low and moderate light intensity (*n* = 6); Figure S4: Experimental (a) and model-based (b) spectra of leaf reflectance in pea plants, which were cultivated for 9 days under the low and moderate light intensity (*n* = 6 for experiments).
