# Supplementary material for: Development of Analytical Model to Describe Reflectance Spectra in Leaves with Palisade and Spongy Mesophyll
Source: Plants (Basel). 2024 Nov 20;13(22):3258. doi: 10.3390/plants13223258 (PMC11598734; doi:10.3390/plants13223258)
Supplement: Supplementary file 1 [file plants-13-03258-s001.zip › Figure S1.pdf]

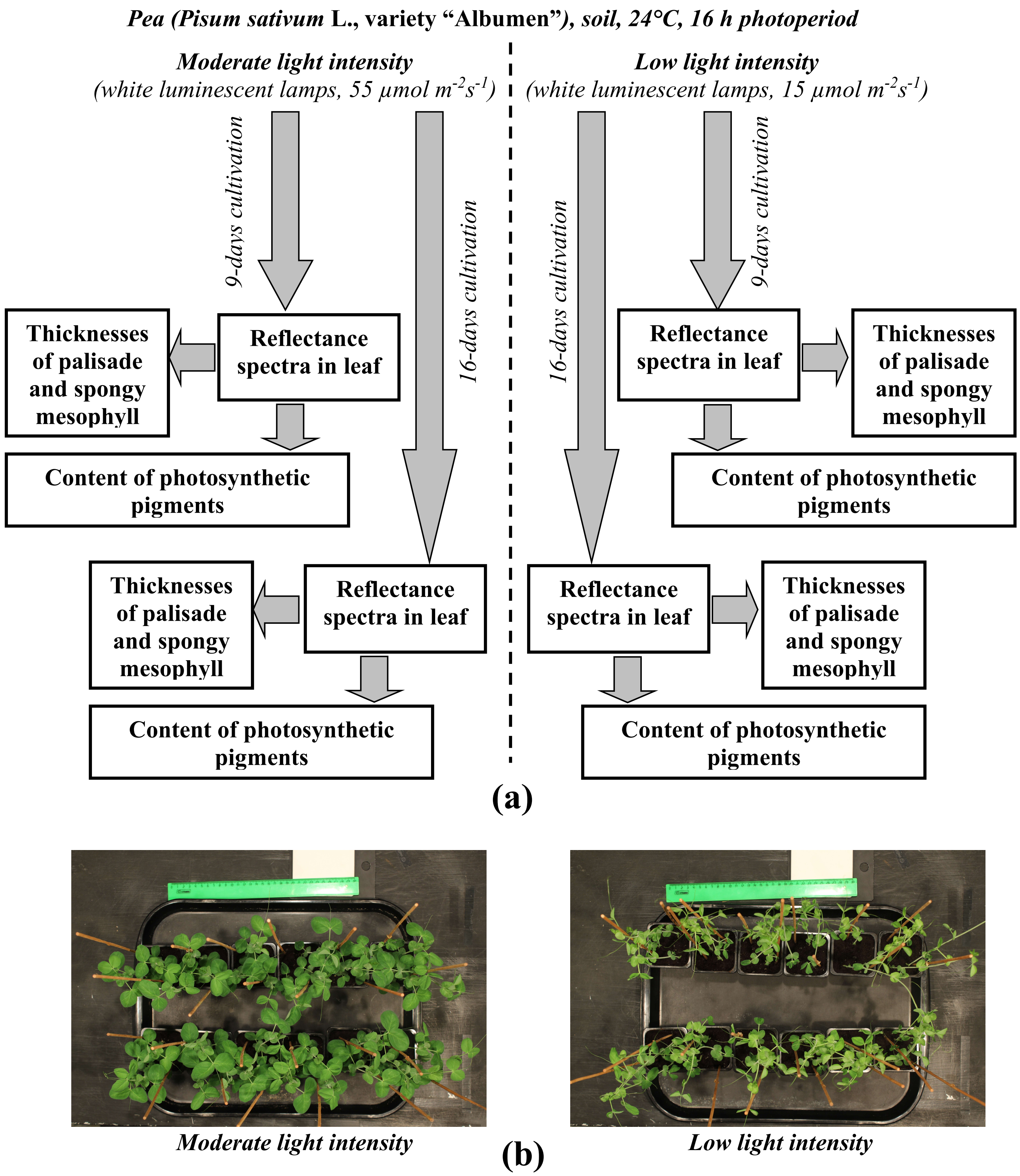

Figure S1. Scheme of cultivation of pea and measurements of its parameters **(a)** and photos of plants, which were cultivated under the moderate and low light intensity **(b)**. Leaf reflectance spectra, content of photosynthetic pigments (chlorophyll a, chlorophyll b, and carotenoids), and thicknesses of palisade and spongy mesophyll were used to parameterize and verify the developed model.
