# Supplementary material for: Development of Analytical Model to Describe Reflectance Spectra in Leaves with Palisade and Spongy Mesophyll
Source: Plants (Basel). 2024 Nov 20;13(22):3258. doi: 10.3390/plants13223258 (PMC11598734; doi:10.3390/plants13223258)
Supplement: Supplementary file 1 [file plants-13-03258-s001.zip › Figure S2.pdf]

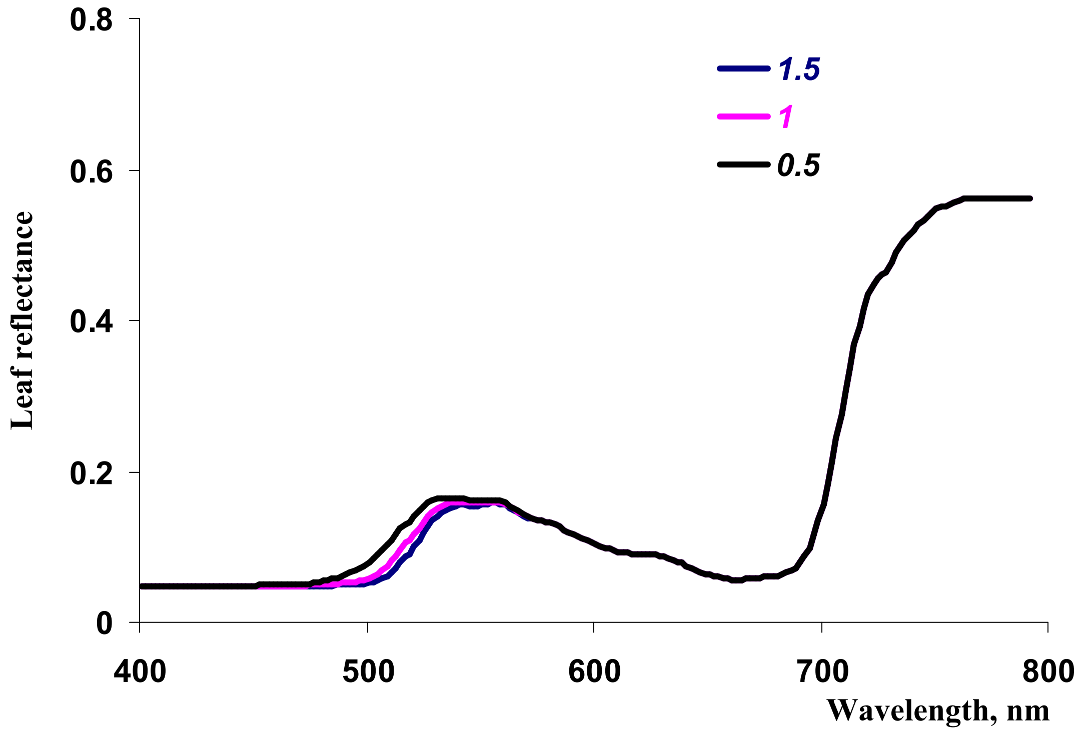

Figure S2. Spectra of leaf reflectance calculated at C_{Car}=1.5 mg cm^{-3}, C_{Car}=1 mg cm^{-3}, and C_{Car}=0.5 mg cm^{-3}. These concentrations correspond to average concentrations of carotenoids in leaf equaling to 0.75, 0.5, and 0.25 mg cm^{-3}. Other parameters of the model are same with the parameters, which were used for simulation of spectrum in Figure 8c.
