# Supplementary material for: Development of Analytical Model to Describe Reflectance Spectra in Leaves with Palisade and Spongy Mesophyll
Source: Plants (Basel). 2024 Nov 20;13(22):3258. doi: 10.3390/plants13223258 (PMC11598734; doi:10.3390/plants13223258)
Supplement: Supplementary file 1 [file plants-13-03258-s001.zip › Figure S3.pdf]

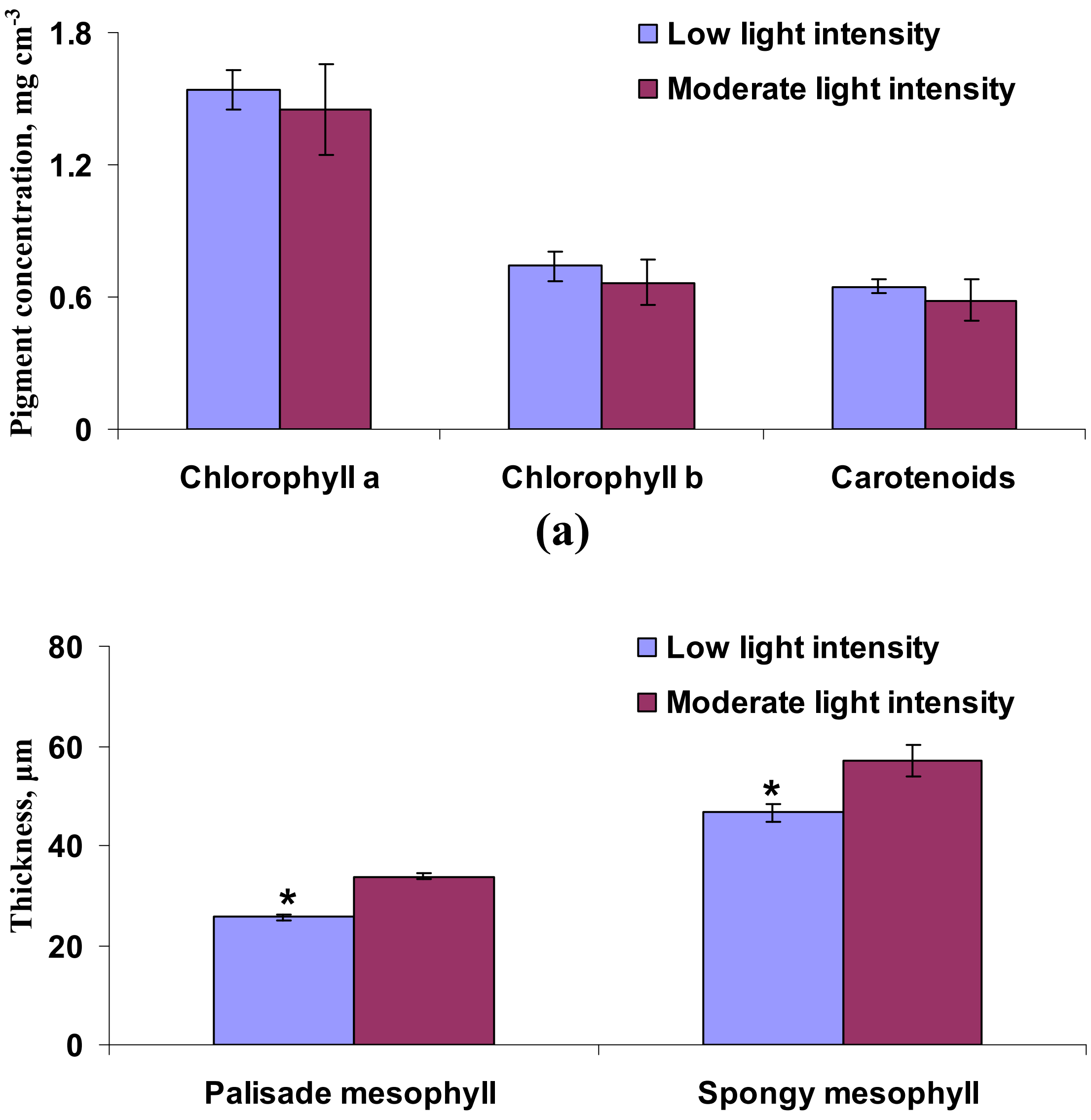

(b)

Figure S3. Experimental concentrations of chlorophyll a, chlorophyll b, and carotenoids **(a)** and thicknesses of palisade and spongy mesophyll **(b)** in second mature leaves of pea plants, which were cultivated for 9 days under the low and moderate light intensity (*n*=6). *, the value is significantly differed from this value in plants, cultivated under the moderate light intensity.
