# Supplementary material for: Development of Analytical Model to Describe Reflectance Spectra in Leaves with Palisade and Spongy Mesophyll
Source: Plants (Basel). 2024 Nov 20;13(22):3258. doi: 10.3390/plants13223258 (PMC11598734; doi:10.3390/plants13223258)
Supplement: Supplementary file 1 [file plants-13-03258-s001.zip › Figure S4.pdf]

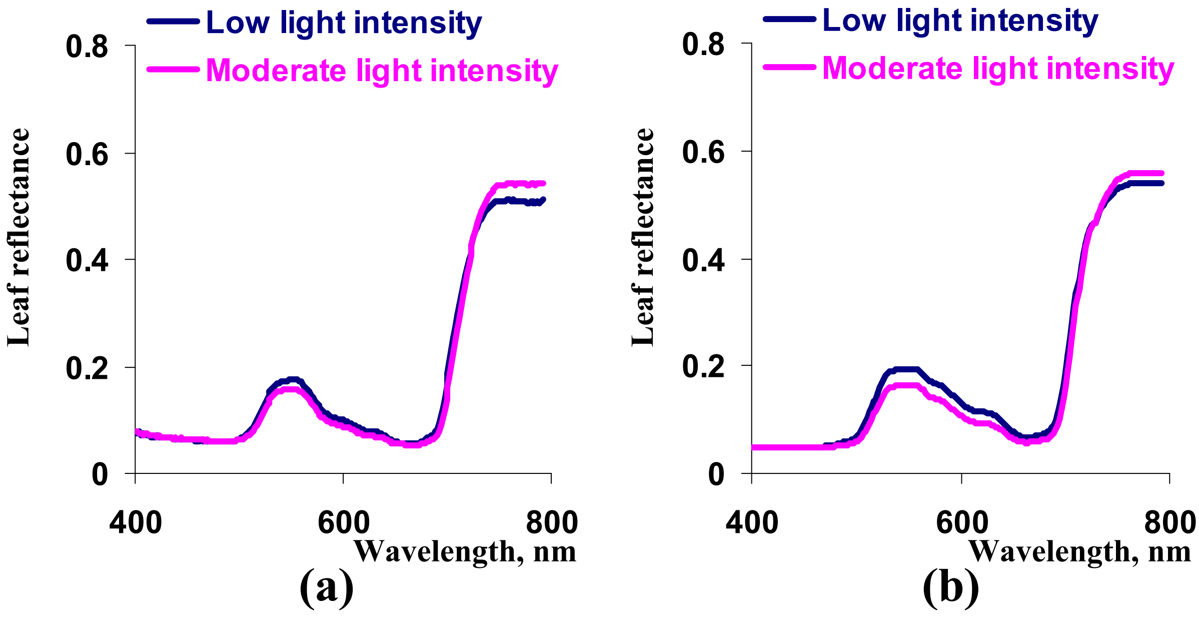

Figure S4. Experimental **(a)** and model-based (**b**) spectra of leaf reflectance in pea plants, which were cultivated for 9 days under the low and moderate light intensity (*n*=6 for experiments). The average experimental reflectance spectrum (*n*=6) measured in the second mature leaf of pea plants (PolyPen RP 410) is shown. Standard errors are not shown because they are small. In variant “Moderate light intensity”, *h*=33.8 µm and *l*=57.1 µm (see Figure S3b); other parameters were not changed. In variant “Low light intensity”, *h*=25.7 µm and *l*=46.6 µm (see Figure S3b); other parameters were not changed. R^2 between experimental and model-based dependences were 0.989 (variant “Moderate light intensity”) and 0.976 (variant “Low light intensity”).
